# Protocol for visual-acoustic intervention with service delivery in-person and via telepractice (VISIT) non-inferiority trial for residual speech sound disorder

**DOI:** 10.1186/s12887-024-05364-z

**Published:** 2025-01-27

**Authors:** Tara McAllister, Jonathan L. Preston, Elaine R. Hitchcock, Nina R. Benway, Jennifer Hill

**Affiliations:** 1 Department of Communicative Sciences and Disorders, New York University, 665 Broadway, Room 602, New York, New York 10012 USA; 2 Department of Communication Sciences and Disorders, Syracuse University, Syracuse, New York USA; 3 Department of Communication Sciences & Disorders, Montclair State University, Bloomfield, New Jersey USA; 4 Department of Electrical and Computer Engineering, University of Maryland, College Park, Maryland USA; 5 Department of Applied Statistics, Social Science, and the Humanities, New York University, New York, New York USA

**Keywords:** Speech sound disorder, Randomized controlled trial, Biofeedback, Telepractice

## Abstract

**Background:**

Residual speech sound disorder (RSSD) is a high-prevalence condition that negatively impacts social and academic participation. Telepractice service delivery has the potential to expand access to technology-enhanced intervention methods that can help remediate RSSD, but it is not known whether remote service delivery is associated with a reduction in the efficacy of these methods. This project will systematically measure the outcomes of visual-acoustic biofeedback intervention when delivered in-person or online.

**Methods/design:**

This project, *Visual-acoustic Intervention with Service delivery In-person and via Telepractice (VISIT)*, aims to treat 76 children in a parallel randomized controlled clinical trial in which children with RSSD will receive visual-acoustic biofeedback treatment either in person or via telepractice. Eligible children will be speakers of American English aged 9–17 years who exhibit RSSD affecting /ɹ/ but otherwise show cognitive-linguistic and hearing abilities within the typical range. All participants will receive twenty sessions of visual-acoustic biofeedback; they will be randomized, with stratification by pre-treatment speech production ability and site, to complete their treatment sessions either in the laboratory setting or at home via telepractice. For the primary outcome measure, blinded listeners will evaluate changes in the perceived accuracy of /ɹ/ production after the end of treatment.

**Discussion:**

By comparing outcomes in children randomized to receive a standard course of biofeedback treatment either via telepractice or in-person, this study will provide evidence-based guidance for clinicians seeking flexible service delivery options for a challenging and prevalent condition.

**Trial registration:**

ClinicalTrials.gov identifier NCT06517225, 07/23/2024. URL: https://clinicaltrials.gov/study/NCT06517225.

**Supplementary Information:**

The online version contains supplementary material available at 10.1186/s12887-024-05364-z.

## Background

Children with speech sound disorder (SSD) exhibit atypical speech patterns that negatively affect intelligibility, posing a barrier to participation in social and academic settings [1]. Delayed speech development typically resolves by 8–9 years old, but 2–5% of speakers experience residual speech sound disorder (RSSD) that persists through adolescence or even adulthood [2–4]. RSSD is often associated with an increased incidence of peer difficulties or bullying [5–7], and the impact on educational, occupational, and mental health outcomes may be lifelong [1, 8, 9]. RSSD is considered particularly challenging to remediate, and speech-language pathologists (SLPs) have called on the research community to develop improved treatment methods for this diagnosis [10]. Residual errors affecting rhotic sounds in North American English (which include /ɹ/ as in the start of the word *read* and /ɝ/ as in the word *her*) are widely regarded as one of the most common and challenging forms of RSSD [10]. To ensure a relatively homogeneous population, this study focuses on individuals with RSSD affecting rhotic sounds.

Recent evidence suggests that visual biofeedback, which uses instrumentation to provide real-time information about aspects of speech that are typically outside the speaker’s conscious control [11], can be used to enhance intervention for RSSD and other speech disorders. Visual biofeedback can incorporate various technologies. The focus in this study is on *visual-acoustic biofeedback*, in which a microphone and software are used to generate a real-time display of the acoustic signal of speech. In the present case, a real-time Linear Predictive Coding (LPC) spectrum is used to represent the resonant frequencies of the vocal tract, or formants. The learner views a model or template representing the formant pattern for correct productions of the target sound and is encouraged to adjust their own output to match the template in the real-time visual display.

Several small-scale studies have documented positive outcomes from visual-acoustic biofeedback treatment in individuals who previously showed minimal, if any, improvement with traditional forms of intervention. (Traditional treatment for RSSD generally involves providing auditory models and verbal descriptions of correct articulator placement, then cueing repetitive motor practice.) One study initially provided traditional motor-based treatment to 11 children with RSSD affecting rhotics, then transitioned to a phase of visual-acoustic biofeedback treatment in a staggered fashion [12]. Only two participants showed a significant response to the initial phase of treatment, while six additional participants showed significant improvements in /ɹ/ production after the transition to biofeedback treatment. Another single-case experimental study of 11 children found a significant interaction between treatment condition and order, suggesting that visual-acoustic biofeedback followed by traditional motor-based treatment yielded significantly larger effect sizes compared to the same treatments provided in the reverse order [13]. Finally, in a single-case randomization study providing both traditional treatment and visual-acoustic biofeedback to seven participants [14], three participants were observed to exhibit a statistically significant advantage for visual-acoustic biofeedback over traditional treatment, and none were found to show a significant advantage in the opposite direction. While these small-scale studies have reported promising evidence, a well-powered randomized controlled trial comparing treatment with and without visual biofeedback is needed to provide a high-quality evidence base to guide clinical decision-making. An earlier study aiming to fill this need was preregistered by our team [15] and is currently in the final stages of data collection.

Despite growing evidence of efficacy, real-world adoption of biofeedback remains limited by barriers such as equipment costs and the need for specialized training. Previous research has suggested that delivery of speech-language services via online telepractice may enable greater specialization among SLPs (e.g., for low-incidence conditions such as cleft lip/palate [16]) because recruitment of clients is not geographically limited. If visual-acoustic biofeedback intervention remains effective when delivered in the telepractice context, remote service delivery could represent a valuable means to provide access to biofeedback services to children with RSSD.

In telepractice delivery of speech services, patients are connected with an SLP for diagnosis and treatment over remote conferencing software. Telepractice has a long history in the SLP profession, where it plays an essential role in providing access to certified SLPs for rural and underserved communities. Adoption of telepractice has been increasing for decades as clients and clinicians recognize its potential for greater flexibility and efficiency (e.g [17]). Periods of confinement during the COVID-19 pandemic saw an explosive increase in SLP adoption of telepractice, and many users who tried telepractice for the first time during the pandemic may continue to opt for this approach due to its convenience [18, 19].

A 2017 systematic review on telepractice [20] reported “limited but promising evidence” that telepractice treatment for speech and language can yield effects comparable to in-person treatment. Positive client and parent satisfaction outcomes have also been documented for pediatric and adult speech therapy [21–23]. By contrast, a 2020 systematic review suggested that treatment for SSD via telepractice may yield smaller effect sizes than in-person treatment [24]. Both the 2017 and 2020 studies covered only a small number of studies (*n* = 7 and *n* = 10, respectively), many with limitations such as small sample sizes or retrospective data collection. Thus, it is not well-established if treatment for SSD is equally effective across service delivery models. Because telepractice is advantageous for other reasons, this is an appropriate context to use *non-inferiority trial* methodology [25] to assess whether any reduction in efficacy exceeds a predefined maximum acceptable difference. In the realm of communication disorders, similar reasoning has motivated non-inferiority trials on telepractice versus in-person treatment for aphasia [26], hypokinetic dysarthria [27, 28], and stuttering [29].

Prior to our research team’s efforts leading up to this preregistered trial, no published work has investigated the efficacy of SSD telepractice with visual-121 acoustic biofeedback intervention. During COVID-19 closures, we conducted a pilot study in which seven school-age participants with RSSD received 10 sessions each of traditional and visual-acoustic biofeedback treatment delivered online [30]. Clinically significant gains were exhibited by 5 of 7 participants, supporting the feasibility of visual-acoustic biofeedback treatment via telepractice. We also executed a single-case experimental study in which four children with RSSD received eight sessions each of traditional and visual-acoustic biofeedback treatment via telepractice in a randomized order [31]. Blinded listeners’ perceptual ratings of /ɹ/ word probes indicated that all four participants responded to the combined treatment package with moderate to large effect sizes. No previous research has systematically measured the effects of visual-acoustic biofeedback treatment delivered with identical procedures online and in-person, as we propose to do.

This study will randomly assign *n* = 76 children to receive a standard course of biofeedback intervention delivered in-person or via telepractice, measuring progress with blinded listeners’ ratings of untreated words produced before and after treatment. We hypothesize that biofeedback treatment delivery via telepractice will not be associated with a reduction in efficacy that exceeds a maximum acceptable value determined a priori. We will also administer a survey before and after treatment to assess changes in participants’ socio-emotional well-being, as well as participants’ and caregivers’ satisfaction with the therapy experience.

## Methods and design

Visual-acoustic Intervention with Service delivery In-person and via Telepractice (VISIT) is a parallel-group prospective randomized controlled non-inferiority trial measuring the efficacy of visual-acoustic biofeedback intervention for RSSD affecting /ɹ/ when delivered online versus in-person. All participants will receive visual-acoustic biofeedback intervention following a standard protocol and schedule; they will be randomly assigned to receive this intervention either in the laboratory setting or via online telepractice. Participant allocation in each group will be stratified by pre-treatment severity, since previous research has identified this variable as an important indicator of subsequent treatment response. Finally, allocation will additionally be stratified by site (Montclair, New Jersey; Syracuse, New York). Although not all participants will be physically present at their allocated site during treatment delivery, they must reside close enough to their study site to be able to participate in-person if randomized to that condition. They will also interact with a site-specific study team, which could potentially be relevant for study outcomes.

We plan to enroll a total of 76 children with RSSD. The power analysis to determine this sample size was based on a comparison of 12 participants from a previous study who received visual-acoustic biofeedback treatment in the in-person setting and 11 participants who received a comparable duration of visual-acoustic biofeedback treatment via telepractice. The participants seen in person had an average change score of 39.8% points (standard deviation = 25.8% points) and the participants seen online had an average change score of 36.8% points (standard deviation = 22.6% points). The population standard deviation of the outcome, after controlling for effects of clinician and baseline accuracy, was estimated at 18% points. Using the formula from Flight & Julious 2016 [32], the sample size of 38 participants per group was computed to achieve 80% power for an alpha level of 5%, assuming the non-inferiority margin delta derived as described under *Data analysis plan*. Power analysis was conducted using an online power calculator for non-inferiority trials [33].

### Recruitment

This study received ethics approval through the Biomedical Research Association of New York (BRANY, protocol #18-10-393). VISIT is a multi-site study with two sites coordinating treatment (Montclair State University and Syracuse University) and a central site responsible for data processing/analysis (New York University). Written assent and permission will be obtained from all study participants and their parent or guardian. REDCap electronic data capture tools [34, 35] hosted at Syracuse University will be used to obtain electronic consent/assent and responses to questionnaires; it will also be used for entry of study data, including scores on eligibility testing and treatment session information. Double-entry and range-restricted data fields will be used for quality control.

Participants will be recruited primarily through referrals from community SLPs, who will be contacted by informational posts to listservs, social media channels, alumni lists, and personal contacts. Other participants may be referred directly by their parent/guardian, who will be contacted through announcements posted in parenting groups on listservs and social media, as well as through flyers displayed in public places such as libraries, schools, and pediatricians’ offices. Participant enrollment began in August 2024.

### Eligibility criteria

All participants must be aged between 9;0 and 17;11 (years; months) at the time of enrollment. Participants must speak English as a dominant or equally dominant language and are required to have begun learning English by age 3, as indicated by parent report. In addition, parent report must indicate that participants hear a rhotic dialect of English (i.e., a dialect of English in which the /ɹ/ sound is pronounced in syllable-final position, such as Mainstream American English in contrast with British Received Pronunciation) from at least one speaker in the home, and that participants have expressed a desire to change the way they pronounce the /ɹ/ sound. Participants must have no history of sensorineural hearing loss or developmental disability (e.g., cerebral palsy, Down Syndrome), per parent report. Additional exclusionary criteria include: history of major brain injury, brain surgery, or stroke in the past year; diagnosis of epilepsy or other neurological disorder with seizure incidents or medication changes in the past six months; or the presence of orthodontia that crosses the palate, such as a palate expander.

Participating families will also be required to meet minimum technology requirements in the home, in the event the participant is randomized to receive treatment via online telepractice. Specifically, participating families must report having access to a laptop or desktop computer, not a tablet or Chromebook, and they must report having a broadband internet connection in the home. In addition, participating families must attest that there is a quiet space in the home for the child to join online study sessions.

During the in-person evaluation session, participants must pass a brief examination of oral structure and function and a pure-tone hearing screening at 20 dB Hearing Level. To rule out language deficits that could interfere with participants’ response to intervention, all participants are required to exhibit language skills broadly within normal limits. This will be established either by a passing score on the Clinical Evaluation of Language Fundamentals-5 (CELF-5) [36] screening measure or by a standard score of at least 80 on the Core Language Index of the CELF-5. Additionally, to limit heterogeneity in participants’ level of severity at baseline, participants are required to score below 30% correct (based on the average across two trained listeners) on a 24-item probe list eliciting /ɹ/ at the word level across a balanced representation of phonetic contexts. The Goldman-Fristoe Test of Articulation-3 (GFTA-3) [37] will be administered for descriptive characterization of participants but will not be used as a criterion for inclusion.

As described in our previous research [15], we will use the Syllable Repetition task [38] and the multisyllabic word task of the LinguiSystems Articulation Test [39] to rule out childhood apraxia of speech (CAS) in participants. Participants who score above the predetermined cutoff representing likely CAS on both tasks will be excluded. Participants who score above the cutoff on only one of these two measures will be administered a maximum performance task as a tiebreaker measure. Participants whose task performance is consistent with signs of CAS as outlined by Thoonen et al. [38–41] will be excluded; participants who score within normal limits will be included.

Both male and female children will be recruited for this study, and no participants will be excluded based on sex/gender or racial/ethnic group. In light of the general population demographics of children with speech sound disorders [4], however, we expect that more males will be referred than females.

### Assessment process

An online screening instrument and follow-up phone call will be used to identify any exclusionary criteria that can be indicated via parent report, such as being outside the age range or having a diagnosis of developmental disability. This call will also provide a detailed description of the study and its requirements, including minimum technology needs and scheduling expectations. Families who pass the phone screening will be invited to participate in an in-person eligibility assessment, 1–2 h in duration. Prior to the eligibility visit, consent and assent instruments will be administered in an online call, and electronic questionnaires will be used to collect detailed information about participants’ health and language history, demographic characteristics, and attitudes toward study participation, as well as to assess the impact of RSSD on the participant’s socio-emotional well-being. The in-person assessment will include the eligibility tasks described in the previous section. Participants will also produce custom probes assessing imitative production of /ɹ/ at the single syllable level (stimulability probe [42]) and non-imitative production of /ɹ/ at the word level and sentence level.

Individuals who meet all eligibility criteria will be asked to return for an additional testing session. This session will gather information about auditory and somatosensory acuity, which we will use to address separate research questions. This session will also allow us to administer additional eligibility testing if any results from the initial testing session were inconclusive. In particular, participants who did not pass the CELF-5 screening measure will complete the full CELF-5 Core Language Index. This additional testing session will also allow for the administration of the maximum performance tasks described above to any participants who passed one but not both of the screening measures used to rule out CAS.

### Group allocation

Participants will be randomly allocated to one of two groups in a 1:1 ratio. The *in-person* group will receive visual-acoustic biofeedback treatment from a clinician in a private room in research space at Syracuse University or Montclair State University, while the *telepractice* group will receive equivalent treatment delivered via videoconferencing. As indicated above, random allocation will be stratified by both baseline accuracy and study site. To determine baseline accuracy, the stimulability probe will be rated by the clinician who administered the evaluation and a clinician at the other study site. Based on these ratings, participants will be categorized as more stimulable (both clinicians score the participant as showing > 0% accuracy in baseline stimulability probe) or less stimulable (at least one clinician scores 0% accuracy in baseline stimulability probe). The study statistician will generate confidential participant treatment assignments in batches, where each batch corresponds to a combination of site (Montclair State University versus Syracuse University) and response category (more versus less stimulable). Within each batch, half of the participants in each of the four combinations of accuracy level and site will be allocated to the in-person condition and half allocated to the online condition. To avoid a situation where study team members can predict the allocation of an upcoming participant based on their knowledge of previous allocations within the batch, batch sizes were randomly chosen from a collection of possible sizes ranging from 16 to 96 (reflecting the fact that the size of each batch needs to be a multiple of eight).

In cases where a participant drops out prior to study completion, we will invite the participant to return for a follow-up assessment after the typical duration of treatment elapses, in order to measure their outcomes in the absence of the treatment. For participants who decline to participate in this follow-up assessment, in the intention-to-treat analysis, we will generate an imputed score based on data from the other participants in the group the missing participant was assigned to. To support this imputation, participants will complete one assessment session at the midpoint of treatment (after treatment session 10). We will also conduct an adjusted per-protocol analysis, described below under *Data analysis*.

### Intervention delivery

All treatment will be provided on an individual basis by a certified SLP member of our research team. Consistency across study SLPs is ensured through a standard training process and ongoing fidelity checks, described below. For ethical reasons, we will not ask participants who are enrolled in outside speech therapy to discontinue these services. Instead, we will ask each participant’s parent/guardian to complete a standard questionnaire describing the nature and frequency of any speech services currently provided to their child. The same questionnaire will be readministered at the midpoint of treatment to capture any changes in outside service delivery over the course of the study.

In both the in-person and telepractice conditions, biofeedback treatment will be provided using staRt, a web application for visual-acoustic biofeedback [43, 44]. Participants in both conditions will wear a Plantronics Poly Blackwire 3225 headset (unidirectional microphone with 100 Hz − 10 kHz frequency response) to capture their voice as input to the staRt web application. For in-person sessions, the participant and study SLP will be seated in front of a desktop computer in a clinic room and will use a browser to access the staRt application. For telepractice, the participant and SLP will meet in a password-protected Zoom videoconference room, with the participant joining from their home using a laptop or desktop computer. The SLP will share a link to a private room in the staRt web application. Transmission of audio data will then be taken over by the staRt web application, while video transmission will continue to occur through the Zoom channel. The real-time linear predictive coding (LPC) spectrum that forms the basis of visual-acoustic biofeedback intervention for /ɹ/ will be computed on the local device of the speaker currently selected as the “hot mic”; the coefficients of the computed spectrum will then be transmitted through a data socket and redrawn in the browser of the other party. This configuration minimizes issues of long latency and low resolution that can be problematic when viewing a biofeedback display via screen-sharing technology.

### Schedule and dosage of intervention

**Fig. 1:**
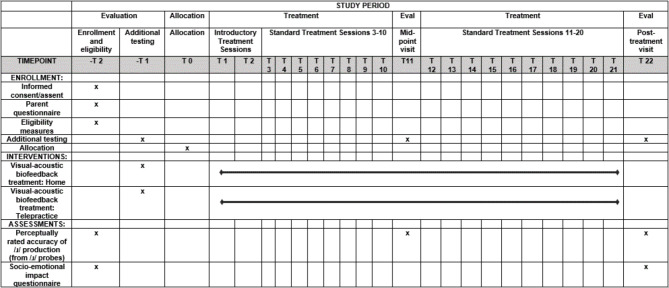
Schedule of evaluation, allocation, treatment, and close-out activities for VISIT

The data collection schedule is outlined in Fig. 1. In both conditions, participants will receive 20 intervention sessions on a roughly semiweekly basis. Intervention sessions will have a target duration of approximately 60 min. The first portion of each session (termed *pre-practice*) will consist of relatively unstructured, highly interactive practice, designed to provide instruction on the phonetic requirements for /ɹ/ and individualized shaping strategies to transform the child’s current productions into perceptually accurate /ɹ/. In the basic therapeutic exchange for pre-practice, the clinician provide an auditory model, elicit an imitation, and provide feedback and cues to increase the accuracy of subsequent attempts [45–48]. Shaping can involve verbal cues for articulator placement or elicitation from facilitative contexts. Suggested cues are summarized in a standard list that is made available through our resources on the Open Science Framework. Visual-acoustic biofeedback will be provided throughout the pre-practice phase. In each session, pre-practice will be discontinued after the participant produces three perceptually accurate productions of all target syllables/words or after ten minutes elapse, whichever comes first.

Pre-practice is followed by the *structured practice* portion of the session, which aims to elicit repetitive motor practice of targets containing /ɹ/ with the goal of making improved production habitual. Structured practice will terminate after the completion of 200 trials containing /ɹ/ (independent of the perceptual accuracy of the trials) or after the total session time reaches 60 min. Each session will aim to elicit at least 150 trials in structured practice, although fewer trials are permissible if necessitated by client or session factors. During structured practice, participants will be cued to produce targets containing /ɹ/ in blocks of 10 trials. Within and between blocks, the clinician will provide quantitative (knowledge of results, or KR) and qualitative (knowledge of performance, or KP) feedback on a fixed schedule as prompted by the staRt software, described in more detail below. Based on research on principles of motor learning, participants will initially complete structured practice in an *Acquisition* mode that provides frequent, detailed feedback with the goal of helping the learner understand the nature of the motor plan for perceptually accurate /ɹ/ [49]. After cumulative accuracy within a session reaches or exceeds 60%, sessions will shift to a *Generalization* mode intended to encourage stabilization of the motor plan and transfer to broader contexts. Additional detail on these two session modes can be found below under *Intervention*.

### Intervention

#### Introductory session 1

*Tongue shapes for /*ɹ*/.* In the first treatment session, participants will receive an initial introduction to articulatory anatomy and tongue shapes for /ɹ/, following a script made available through our resources on the Open Science Framework; completing the script takes roughly 20–25 min. Following this script, the treating clinician will use diagrams and magnetic resonance (MR) images to teach participants to identify different components of the tongue, namely the root, back, blade, and tip. The rationale for this training is that more precise knowledge of anatomy can help learners understand and respond to cues for articulator placement. Participants will then be familiarized, via MR images and line drawings/animations, with the most salient characteristics of tongue shapes for perceptually accurate /ɹ/. They will be told that different speakers use different tongue shapes for /ɹ/, but a few tongue shape properties are shared across perceptually correct /ɹ/ sounds. These include (a) elevation of the anterior tongue, (b) retraction of the tongue root, and (c) bracing of the sides of the tongue against the rear molars/margins of the posterior palate, forming a groove down the midline of the tongue. To verify comprehension, participants will be asked to describe the articulatory components of /ɹ/ and choose between pairs of images representing correct and incorrect tongue shapes for /ɹ/. After the initial instructional period, the clinician will attempt to elicit correct /ɹ/ in a pre-practice phase up to 15 min in duration. No biofeedback will be provided during this training.

Visual aids representing correct articulation of /ɹ/, including MR images and animations, can be made available throughout the course of treatment at the clinician’s discretion. An image judged appropriate for an individual participant’s production of /ɹ/ can be shared at the start of each session and referenced again as needed.

#### Introductory session 2

*Visual-acoustic biofeedback*. In the second treatment session, participants will be oriented to the staRt software used for visual-acoustic biofeedback in a training with a duration of roughly 20–25 min. The staRt software analyzes speech input with an LPC algorithm that is updated in real-time to reflect changes over the course of articulation. The acoustic hallmark of American English /ɹ/ is a lowered frequency of the third formant, F3, and a reduced distance between the second and third formants (F3-F2 distance). In staRt, an adjustable slider superimposed over the dynamic LPC spectrum is used to set a target for F3. The clinician and the participant will jointly complete a scripted tutorial that combines verbal explanations and models with opportunities to interact with the software. The tutorial will begin by introducing sounds other than /ɹ/, such as “ee” and “ah.” It will draw the learner’s attention to differences in the location of the formants (“peaks” in the “wave”) for these sounds, then cue the learner to produce these sounds and compare their peaks to a model. The target formant configuration for /ɹ/ will subsequently be introduced with static images and live demonstrations. To check comprehension, participants will be asked to differentiate between correct and incorrect /ɹ/ as seen in the visual-acoustic display. After the introduction to biofeedback, the clinician will engage the participant in pre-practice for up to 15 min. This pre-practice will resemble the pre-practice phase from the first treatment session, but the biofeedback display will be made available.

For each participant, the adjustable slider that acts as a target for F3 will initially be set to a value for the participant’s age and sex derived from published reference data [50]. If the child starts to achieve perceptually accurate /ɹ/ in treatment, the target frequency for the slider can be updated to match its location in the participant’s own best approximation of /ɹ/. This custom target location will be saved to the participant’s profile for use in subsequent sessions and can be updated as further progress is made.

#### Targets of practice

Articulation of the /ɹ/ sound is influenced by position in the syllable and phonetic context, and children who produce distortions of /ɹ/ may show higher accuracy in some contexts than others [12]. In this study, treatment sessions will elicit /ɹ/ practice in six major contexts laid out in Table 1. These contexts are represented with an equal number of items in the word probe and syllable probe measures administered to assess participant progress.

**Table 1:** Sample words representing six contexts for /ɹ/ elicited in all probes and treatment sessions

Context	Onset position	Non-onset position
**Front vowel context**	*red*,* ran*	*deer*,* chair*
**Central vowel context**	*rug*,* ride*	*bird*,* water*
**Back vowel context**	*rob*,* rude*	*star*,* door*

In the Acquisition mode, participants will practice a fixed list of six syllables, with one syllable representing each context: *ray*,* rye*,* rah*,* ear*,* are*,* er* ([ɝ]). The same syllables will be targeted during pre-practice and structured practice. For participants who advance to Generalization mode, two word representing each of the six contexts will be randomly drawn from a larger word list. These words will be used in pre-practice and at the start of structured practice, with a possibility of advancing to more challenging words (see *Adaptive Difficulty*).

#### Scoring and feedback

In both Acquisition and Generalization modes, participants will practice /ɹ/ in blocks of ten consecutive trials targeting the same syllable or word. Stimulus words will be presented in the staRt software and accompanied by a clinician model at the beginning of each block of 10. The clinician will use a keypress (1 or 0) to score each attempt produced by the participant, using a strict standard where only fully correct productions will receive a score of 1 and distorted productions will receive a score of 0.

After each trial in structured practice, the staRt software will prompt the clinician to provide KP feedback, KR feedback, or no feedback, according to a predetermined schedule. In the Acquisition mode, the clinician will be prompted to provide qualitative KP feedback after every other trial. As in our previous research [15], KP is operationalized as including three elements. First, the clinician must reference what the child is doing or should be doing with the articulators (e.g., “Remember to keep the sides of your tongue up for /ɹ/”). Second, if the biofeedback display was available on the trial in question, the clinician’s feedback must make reference to the visual display. Finally, the clinician is expected to provide a verbal model of correct production for the next trial in the block. In the Generalization mode, the clinician will be prompted to provide a mix of KP and KR feedback, with the frequency of each feedback type changing across the levels of adaptive difficulty described below. When a trial is selected for KR feedback, the staRt software will automatically display a feedback message based on the score entered by the clinician. This feedback may also be verbalized at the clinician’s discretion.

#### Adaptive difficulty

During the structured practice portion of the session in Generalization mode, stimuli will be presented with adaptive difficulty based on the scores entered by the treating clinician. Our rationale for adaptive difficulty, drawing on previous motor learning research [51, 52], is that opportunities for learning during speech practice are maximized when learners practice at a “challenge point” that is neither too hard nor too easy. The hierarchy for adaptive difficulty is modified from our previously published work [53]. After each block of ten trials, the software automatically tallies the clinician’s accuracy ratings and makes a determination regarding task difficulty for the next block. If the participant’s accuracy in the previous block was 80% or higher, the next block will step up to the next difficulty level; if accuracy was 50% or lower, the next block will drop to a lower difficulty level; otherwise, difficulty will hold steady at the current level. The parameters used to manipulate task difficulty include the linguistic complexity of the utterance used to elicit /ɹ/, the frequency with which verbal feedback and/or biofeedback are provided, and the mode of elicitation (such as imitation versus independent reading). These parameters will be adjusted on a rotating basis, resulting in a total of 17 levels of difficulty that are listed in Table 2. The parameter settings are saved at the end of each session and used as the starting point for the participant’s next treatment session.

**Table 2:** Levels of adaptive difficulty implemented in the staRt software. Parameters (represented in columns) change on a rotating basis between levels; the parameter that was changed in a given level is in bold

Level	Complexity	Feedback Frequency	Mode of Elicitation
1	Syllable	3 KR, 2 KP + model; biofeedback at 80%	Read independently
2	**1 syllable word**	3 KR, 2 KP + model; biofeedback at 80%	Read independently
3	1 syllable word	3 KR, 2 KP + model; **biofeedback to 50%**	Read independently
4	1 syllable word	3 KR, 2 KP + model; biofeedback at 50%	**Blocked prosodic manipulation**
5	**1 syllable word with competing /l/ or /w/**	3 KR, 2 KP + model; biofeedback at 50%	Blocked prosodic manipulation
6	1 syllable word with competing /l/ or /w/	3 KR, **1 KP + model;** biofeedback at 50%	Blocked prosodic manipulation
7	1 syllable word with competing /l/ or /w/	3 KR, 1 KP + model; **biofeedback to 20%**	Randomized prosodic manipulation
8	1 syllable word with competing /l/ or /w/	3 KR, 1 KP + model; biofeedback at 20%	**Randomized prosodic manipulation**
9	**2 syllable word**	3 KR, 1 KP + model; biofeedback at 20%	Randomized prosodic manipulation
10	2 syllable word	**2 KR**, 1 KP + model; biofeedback at 20%	Randomized prosodic manipulation
11	2 syllable word	2 KR, 1 KP + model; **biofeedback to 0%**	Randomized prosodic manipulation
12	**2 syllable word with competing /l/ or /w/**	2 KR, 1 KP + model; biofeedback at 0%	Randomized prosodic manipulation
13	2 syllable word with competing /l/ or /w/	**2 KR with self-evaluation;** 1 KP + model; biofeedback at 0%	Randomized prosodic manipulation
14	**Words in carrier phrases**	2 KR with self-evaluation; 1 KP + model; biofeedback at 0%	Randomized prosodic manipulation
15	**Words in sentences**	2 KR with self-evaluation; 1 KP + model; biofeedback at 0%	Randomized prosodic manipulation
16	**Sentences with multiple /r/ targets**,** clinician scores only one**	2 KR with self-evaluation; 1 KP + model; biofeedback at 0%	Randomized prosodic manipulation
17	**Sentences with multiple /r/ targets**,** clinician scores both**	2 KR with self-evaluation; 1 KP + model; biofeedback at 0%	Randomized prosodic manipulation

### Clinician training

To ensure that study clinicians have adequate knowledge to support effective treatment delivery, all treating clinicians will be required to review a series of informational modules in Powerpoint format. These modules were developed for our previous preregistered trial [15] and updated for the current study. A total of five separate modules cover the following topics: how /ɹ/ is produced, how to cue the /ɹ/ sound with articulator placement cues, an overview of visual-acoustic biofeedback, how to cue the /ɹ/ sound with visual-acoustic biofeedback in staRt, and a guide to the adaptive difficulty hierarchy built into the staRt software. Treating clinicians will meet individually with the principal investigator at their site after completing the training modules in order to discuss and resolve any questions or points of confusion.

### Treatment fidelity

Clinicians’ adherence to standard protocols will be assessed by reviewing screen-recorded video and audio from a selection of sessions for each participant. To encourage a uniform standard across clinical sites and preserve blinding at the central site, clinicians from the two sites will perform fidelity checks for one another. Two treatment sessions from each half of the study (before and after the midpoint visit) will be randomly selected for fidelity checking for each participant. Because of the large number of trials elicited in each treatment session, fidelity checks will cover a randomly selected 50-trial subset of the selected sessions.

During each fidelity check, a clinician from a different site will review the screen-recorded video of the selected session and compare it to an output record detailing trial-by-trial prompts generated by the staRt software. For each trial, the staRt output includes information on: (1) whether biofeedback was expected to be provided or withheld; (2) whether a verbal model from the clinician was expected before the trial; (3) whether KP feedback was expected after the trial, (4) whether KR feedback was expected after the trial, and (5) whether the client should have been prompted to evaluate the accuracy of their own production. In each case, the clinician performing the fidelity check will indicate whether the treating clinician’s behavior in the recording aligns with the expected behavior indicated in the software output. If KP feedback was indicated, the checking clinician will additionally report whether the treating clinician’s verbal feedback included the three components outlined above: reference to the target articulatory behavior, reference to the visual biofeedback display, and provision of a verbal model.

### Recording and equipment

For evaluation sessions, each site will use 64-bit PCs operating Windows 10 or 11 with relevant software. All audio recordings from evaluation sessions will be obtained with a head-mounted microphone (AKG C520 Professional Head-Worn Condenser microphone) positioned so the microphone arm is perpendicular to the corner of the mouth. The audio signal from the head-mounted microphone will be routed to the PC through an audio interface (Focusrite Scarlett 2i2 or Behringer UMC 404HD). The primary recording of each session activity will be registered in lossless FLAC audio extracted from mkv screen-recorded video generated by Open Broadcaster Software (OBS). An additional line out from the audio interface to a solid-state digital recorder will register a backup copy of the audio in the event of any issue with the primary recording. To accommodate individual differences in vocal volume, gain settings on the audio interface can be adjusted within a predetermined range. All recordings will be registered using a 44,100 Hz sampling rate and 16-bit encoding.

For treatment sessions, participants in both conditions will wear the Plantronics Poly Blackwire 3225 headset mentioned previously. The headset will be connected to the computer used for sessions, which will be a desktop PC for participants who receive in-person treatment and the participating family’s home device (laptop or desktop computer) for participants in the online condition. The session will be recorded in lossless FLAC audio extracted from mkv screen-recorded video generated by OBS software with 44,100 Hz sampling rate and 16-bit encoding.

### Outcomes measurement

For our primary outcome measure, we will evaluate change in /ɹ/ production accuracy by obtaining perceptual ratings of /ɹ/ production accuracy in the word probe elicited in the pre-treatment evaluation visit and again in a post-treatment assessment visit scheduled within approximately one week of the end of treatment. The word probe consists of 24 words divided evenly across the six /ɹ/ contexts targeted in treatment; to assess generalization, the probe words do not overlap with the word lists targeted in treatment. As in our previous research [15], we will obtain perceptual ratings of /ɹ/ production accuracy from untrained listeners recruited via online crowdsourcing. Raters will be required to report speaking American English since early childhood and must originate from US-based IP addresses. Each word probe recording will be split into word-level productions. These word-level recordings will be pooled across speakers and time points and presented in a randomized order for rating. Raters will see the orthographic representation of each word and will be asked to assign a binary rating (correct/incorrect) to the /ɹ/ sound in each word. Based on the results of our previous methodological research [54, 55], we will collect ratings until at least 9 unique listeners have rated each token. Our primary measure of the accuracy of /ɹ/ production will be the proportion of “correct” ratings out of the total number of ratings, which has been found to correlate strongly with acoustic measures as well as expert listeners’ ratings of /ɹ/ [54, 55].

As a secondary outcome measure, a survey assessing the social-emotional impact of RSSD will be administered to participants and their parents in the initial evaluation and post-treatment assessment [5]. The survey includes 11 items (e.g., “My speech/my child’s speech sounds different from the speech of other children my/their age”; “My speech/my child’s speech has an impact on my/their academic performance”) that are presented with the response options “yes,” “no,” and “sometimes.” Responses will be analyzed using a Generalized Partial Credit Model [56], which combines individual item scores into an overall impact score. Each item is weighted to reflect its stronger or weaker association with the total score.

### Data analysis plan

The first statistical consideration for this study is the value of the margin delta (Δ), which represents the maximum acceptable loss of effect for an alternative treatment relative to an established one in a non-inferiority trial. Previous literature has suggested that delta may be defined as “less than the minimum difference of clinical interest (i.e., the minimum clinically important difference; MCID)” [28, 57]. We defined the MCID with reference to a meta-analysis of eleven single-case experimental studies of biofeedback intervention for RSSD previously published by our team [58]. In this meta-analysis, we generated de-identified plots of participants’ accuracy before and after treatment, based on perceptual ratings aggregated over blinded listeners. Three experienced researchers visually inspected these plots and reached a consensus judgment classifying each participant as responding or failing to respond to treatment. This “gold standard” binary classification was then used to compute sensitivity, specificity, and combined sensitivity-specificity for various cutoff values. ROC curve analysis revealed that a raw mean difference of 13.9% points represented the optimal cutoff between responder and non-responder categories (optimal combined sensitivity-specificity using Youden’s J). We adopt this cutoff value as the MCID. However, it is recommended that Δ be set to a value less than the MCID. Previous non-inferiority trials in the speech context have considered different values, including half of the MCID [27] or 75% of the MCID [28]. Based on the argument that high variability in response to speech treatment may lead to large confidence intervals [28], we will set our value of delta to 75% of the MCID, or 10.4% points.

The primary statistical model described below will be fit twice, once following the intention-to-treat principle, with imputed scores generated for cases of attrition, and once in an adjusted per-protocol analysis; see below for additional detail. Assessment of outcomes following both approaches is recommended for non-inferiority trials [25]. If the lower bound of the 95% confidence interval centered on the observed difference falls above the noninferiority margin of -10.4 in both models, we will conclude that noninferiority has been established.

In the intention-to-treat analysis, we will impute an outcome for participants who drop out of the study and are lost to follow-up based on data from the individuals who have complete data and are in the same treatment assignment. In the per-protocol analysis, we will attempt to estimate the effect of telehealth versus in-person treatment only for those in the sample who remained in the trial and completed all assessments. Because estimates of this effect can be biased due to systematic differences that arise when focusing on this non-random sample from the trial, we will perform an *adjusted* per-protocol analysis using inverse probability of treatment weighting, following recommendations from recent methodological research [59, 60].

Our primary outcome measure of interest is change in perceptually rated accuracy of /ɹ/ sounds from pre- to post-treatment, with perceptual ratings of pre- and post-treatment probes obtained from blinded listeners as described above. We will compute this change score for all participants and will use a linear model with a fixed effect of group to compute the 95% confidence interval around the difference in change scores between groups. The in-person group will be treated as the reference level, so that a negative value indicates smaller gains when treatment is delivered via telepractice versus in-person. The model will also control for the randomization strata of site and baseline severity group (less stimulable versus more stimulable), as well as baseline accuracy as a continuous predictor. Lastly, we do not plan to include a fixed effect of age because our previous research has not supported such an effect (e.g [61]). However, we will test for a correlation between age and treatment outcomes and if a statistically significant correlation is present, we will include age as a controlled covariate.

A secondary analysis will report pre- to post-treatment changes on the 11-item survey assessing the social, emotional, and academic consequences of RSSD that will be administered to participants and their parents both before and after the course of treatment. We will model impact score (as defined in [5]) as the outcome variable with fixed effects of time, treatment group, and the time by treatment group interaction, as well as clinician and initial accuracy level. Likelihood ratio tests will be used to assess the significance of fixed effects and interactions in the final model. A significant effect of time would suggest a change in the perceived impact of RSSD over the course of treatment, while a significant interaction between time and treatment group could indicate a difference in the magnitude of the change in perceived impact across treatment conditions.

## Discussion

### Potential significance

When the proposed data collection is complete, we will have measured changes in /ɹ/ production accuracy, as well as patient-reported outcomes, in children randomized to receive a standard course of biofeedback treatment either via telepractice or in-person. This study will fill a need for high-quality evidence to guide clinical practice as the SLP field shifts toward greater use of telepractice [18, 19]. Developing engaging, evidence-based materials for online use has been identified as one of the most significant challenges for telepractice service delivery [62]. This study aims to scientifically validate an interactive tool for biofeedback treatment via telepractice that is widely available to practicing SLPs. Evidence for the efficacy of biofeedback in the telepractice context could be particularly valuable because children who would otherwise be unable to access biofeedback services may gain access through remote providers.

### Potential limitations

It is important to acknowledge the limitations of the present study. Non-inferiority trials are known to pose a number of challenges for causal inference. First, the definition of the “minimum clinically important difference” is inherently subjective, and there is a lack of agreement in the literature on what criteria to use when setting the parameter Δ (e.g., half of the MCID [27] or 75% of the MCID [28]). We set Δ to 10.4% points based on our reanalysis of previously completed studies, but we acknowledge that other researchers could review the same data and draw a different conclusion about the minimum bound for a clinically meaningful difference between groups.

A second limitation of the non-inferiority design is the potential for non-adherence to the proposed treatment package to influence the conclusions of the study. In our research, the most common form of non-adherence is early study termination. If the types of study participants who terminate early in one condition are different from those that terminate early in the other, the intention-to-treat analysis might provide an overly optimistic assessment regarding non-inferiority. However, if these differences are explainable by our observed covariates, our adjusted per-protocol estimate could help remedy this situation. In accordance with recommendations for non-inferiority studies, we intend to report the results of the VISIT trial using both intention-to-treat and per-protocol analyses. For a strong conclusion of non-inferiority, both methods must show no difference between the treatment conditions exceeding the margin Δ. The US Food and Drug Administration recommends “close examination” of results where a discrepancy is observed between the intention-to-treat and per-protocol analyses. For example, consider a hypothetical scenario where the true effect size of treatment is smaller in the online condition than the in-person condition, but attrition is higher in the in-person condition, which could arise if participants find it more challenging to comply with the prescribed treatment schedule when visits are in-person. This could manifest as a difference in outcomes between the intention-to-treat analysis and the per-protocol analysis, with the latter showing a difference between treatment conditions and the former showing none. While such a finding would not support a strong conclusion of non-inferiority, it could still provide valuable information about real-world strengths and limitations of different service delivery methods. The inclusion of such results in the evidence base could help clinicians and clients make an informed decision based on their own personal values and needs.

An additional limitation of the present study is the possibility that either clinicians or participants/parents could carry their own preference for one service delivery context over another. While we have made every effort to maintain equipoise in our study planning discussions and in how we represent the study to potential participants, there is no way to completely eliminate individual bias. However, we consider information about participant and family preferences to be inherently valuable. We will administer surveys to collect this information in a systematic way and will take these expressed preferences into consideration alongside evidence of efficacy in our interpretation of outcomes.

Finally, some limitations of the generalizability of the findings of this study should be acknowledged. Our primary outcome measure assesses /ɹ/ production accuracy at the word level, rather than at higher levels (sentence or conversation) that might be more representative of learners’ ability in naturalistic communicative situations. The primary outcome measure also evaluates accuracy within a few days of the final treatment session, rather than evaluating the maintenance of gains on a longer time level. However, we do plan to measure participants’ performance six weeks after the end of treatment as part of a separate but related study (not described here) investigating the maintenance of gains made through biofeedback intervention. Lastly, the conditions of treatment delivery in the present study – up to two hours of individual treatment per week – are not reflective of real-world practice patterns for the great majority of SLPs. In the school setting, for instance, SLPs are likely to see children with speech goals in group rather than individual sessions, and the number of minutes of treatment per week is likely to be lower. Our goal in the present study is to measure the efficacy of treatment in a somewhat idealized context; in future research, we hope to shift our focus to the effectiveness of treatment delivered under more realistic conditions.

## Electronic supplementary material

Below is the link to the electronic supplementary material.

Supplementary Material 1

## Data Availability

https://osf.io/wysv9/.

## Abbreviations

RSSD: Residual speech sound disorder
SLP: Speech-language pathologist
KR: Knowledge of results (feedback)
KP: Knowledge of performance (feedback)
LPC: Linear predictive coding (spectrum)
MR: Magnetic resonance (imaging)
CAS: Childhood apraxia of speech
CELF-5: Clinical Evaluation of Language Fundamentals - Fifth Edition
GFTA-3: Goldman -Fristoe Test of Articulation - Third Edition
OBS: Open Broadcaster Software
FLAC: Free lossless audio codec
MCID: Minimum clinically important difference
staRt: Speech therapist’s app for “R” treatment
BRANY: Biomedical Research Association of New York
Hz: Hertz
kHz: Kilohertz
PC: Personal computer
